# A model of the circadian clock in the cyanobacterium Cyanothece sp. ATCC 51142

**DOI:** 10.1186/1471-2105-14-S2-S14

**Published:** 2013-01-21

**Authors:** Nguyen Xuan Vinh, Madhu Chetty, Ross Coppel, Sandeep Gaudana, Pramod P Wangikar

**Affiliations:** 1 Gippsland School of Information Technology, Monash University, Australia; 2 Department of Microbiology, Monash University, Australia; 3 Chemical Engineering Department, Indian Institute of Technology, Bombay, India

## Abstract

**Background:**

The over consumption of fossil fuels has led to growing concerns over climate change and global warming. Increasing research activities have been carried out towards alternative viable biofuel sources. Of several different biofuel platforms, cyanobacteria possess great potential, for their ability to accumulate biomass tens of times faster than traditional oilseed crops. The cyanobacterium *Cyanothece *sp. ATCC 51142 has recently attracted lots of research interest as a model organism for such research. *Cyanothece *can perform efficiently both photosynthesis and nitrogen fixation within the same cell, and has been recently shown to produce biohydrogen--a byproduct of nitrogen fixation--at very high rates of several folds higher than previously described hydrogen-producing photosynthetic microbes. Since the key enzyme for nitrogen fixation is very sensitive to oxygen produced by photosynthesis, *Cyanothece *employs a sophisticated temporal separation scheme, where nitrogen fixation occurs at night and photosynthesis at day. At the core of this temporal separation scheme is a robust clocking mechanism, which so far has not been thoroughly studied. Understanding how this circadian clock interacts with and harmonizes global transcription of key cellular processes is one of the keys to realize the inherent potential of this organism.

**Results:**

In this paper, we employ several state of the art bioinformatics techniques for studying the core circadian clock in *Cyanothece *sp. ATCC 51142, and its interactions with other key cellular processes. We employ comparative genomics techniques to map the circadian clock genes and genetic interactions from another cyanobacterial species, namely *Synechococcus elongatus *PCC 7942, of which the circadian clock has been much more thoroughly investigated. Using time series gene expression data for *Cyanothece*, we employ gene regulatory network reconstruction techniques to learn this network *de novo*, and compare the reconstructed network against the interactions currently reported in the literature. Next, we build a computational model of the interactions between the core clock and other cellular processes, and show how this model can predict the behaviour of the system under changing environmental conditions. The constructed models significantly advance our understanding of the *Cyanothece *circadian clock functional mechanisms.

## Background

Cyanobacteria are one of the most primitive forms of plant, with their mechanism of photosynthesis similar to that of higher plants. However they are in fact much more efficient converter of solar energy thanks to their simple cellular structure. It has been reported that some cyanobacteria can accumulate biomass as much as 30 times more efficient than traditional oilseed crops such as corn and soybeans (as per dried biomass/area/year), and as such has attracted lots of research interest for being a viable biofuel platform. *Cyanothece *sp. ATCC 51142, hereafter *Cyanothece*, has recently gained increasing attention. This unicellular cyanobacterial strain is involved not only in photosynthesis but also in nitrogen fixation within the same cell. As a byproduct of nitrogen fixation, *Cyanothece *has been recently shown to produce biohydrogen at very high rates that are several folds higher than previously described hydrogen-producing photosynthetic microbes [1]. Since the key enzyme for nitrogen fixation is very sensitive to oxygen produced by photosynthesis, *Cyanothece *employs a sophisticated temporal separation scheme, where nitrogen fixation occurs at night and photosynthesis at day. At the core of this temporal separation scheme is a robust clocking mechanism--the circadian clock.

Until recently, cyanobacteria were the only prokaryotes reported to possess circadian rhythmicity [2]. Organisms rely on the circadian clock to plan ahead their actions for maximal efficiency. For example, just before the light period, some of the cellular processes for photosynthesis are already activated and ready for functioning. It is the same for the dark period in nitrogen-fixing species like *Cyanothece*, where it has been observed that just before entering the dark period, a large amount of energy has been mobilized in a ready-to-use form, ready for Nitrogen fixation which is an energy-intensive process [3]. For its ease of genetic manipulation, the cyanobacterium *Synechococcus elongatus *PCC 7942, hereafter *S. elongatus*, is widely chosen as a model organism for cyanobacterial circadian clock studies [4-6]. Compared to other cyanobateria that also possess a circadian clock but do not perform nitrogen fixation such as *S*. *elongatus*, the circadian clock of *Cyanothece *is likely more complex and may involve more input/output pathways to coordinate the tight regulation of photosynthesis and nitrogen fixation pathways. Also, compared to the *S. elongatus *circadian clock which has been quite thoroughly investigated and reported in the literature, *Cyanothece *circadian clock has only very recently received a few preliminary investigations. In [7], using time series gene expression data, Wang et al. characterized *Cyanothece *genes and cellular processes that oscillate in a circadian rhythm. They found that nearly 30% of genes (i.e., ~1500 genes) have a significant rhythm with 24 h period. To find out to what extent the core clock explains the oscillation of other genes, they built an oscillatory network including one master clock and three Kuramoto-type peripheral oscillators. The phase variables of the peripheral clocks were then used to reproduce expression patterns of circadian-clock controlled genes. Although this interesting study showed that their model can relatively faithfully reproduce the gene expression patterns, it lacks connections to the current literature on circadian clock study in our opinion. In particular, the knowledge on input/output genes and pathways previously reported for *S. elongatus *was not refereed to and compared against. In another recent related study [8], McDermott et al. built a predictive model of *Cyanothece *gene regulatory network, in which transcription factors and network bottlenecks were found to be strong predictors of system behaviour. The focus of that research however was not to elicit the interactions between the circadian clock and other genes and pathways.

In the current paper, we set out to study the circadian clock in *Cyanothece*. In particular, we first use comparative genomics to find the homology between the core circadian clock genes in *S*. *elongatus *and *Cyanothece*. The genetic interactions within the circadian clock of *S. elongatus *are then extrapolated to *Cyanothece*. Next, to partly verify these interactions, we use *Cyanothece *time series expression data and network reconstruction techniques to reconstruct the clock *de novo*. The reconstructed network is then compared against the extrapolated interactions. Finally, we build a predictive model between the clock genes and other key cellular process regulators.

Our model shows that the putative clock genes found a good predictor set of the system behaviour, even in changing environmental conditions. The result of this study suggests that the extrapolated information is highly informative, and significantly advances our understanding of the *Cyanothece *circadian clock.

### Review on Synechococcus elongatus PCC 7942 circadian clock

**Figure 1:**
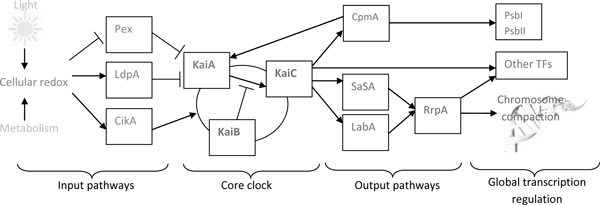
**A schematic diagram of the *Synechococcus elongatus *PCC 7942 circadian clock, comprising of input pathways, the core clock genes, output pathways and other transcription factors/metablic genes**.

In this section, we briefly review the recent literature on *S. elongatus*--a model organism for circadian clock study. A schematic diagram of the *S. elongatus *circadian clock is presented in Figure 1. The core of this clocking mechanism is built upon three proteins, KaiA, KaiB and KaiC. Using only these three purified proteins, together with energy, in the form of ATP/ADP, it is possible to reconstitute in vitro an oscillator with period of roughly 24 h [4]. The oscillation of the clock is created via the ordered phosphorylation and de-phosphorylation of the KaiC protein, facilitated by the KaiA and KaiB proteins. KaiC is both an autokinase and autophosphatase that can be phosphorylated at two positions, serin 431 and threonine 432 [5]. It can have four possible phosphorylated states: full at both S431 and T432 (ST-KaiC), S431 only (S-KaiC), T432 only (T-KaiC), and unphosphorylated (U-KaiC). It is known that the phosphorylation cycle of KaiC occurs in the following order: U-KaiC → T-KaiC → ST-KaiC → S-KaiC → U-KaiC. Naturally, KaiC's autophosphatase activity dominates its autokinase activity. KaiA shifts the equilibrium towards autokinase, while KaiB negates KaiA's action, by inactivating KaiA and thus shifting the equilibrium towards autophosphatase activity [5]. Although a simple clock based on only three purified Kai-A, B, C proteins can be reconstituted *in vitro *in the absence of transcription and translation, this oscillator stops working at 20°C. In contrast, the *in vivo *oscillators still operate robustly under the same condition. This suggests that input transcription and translation play a role in making the clock robust to environmental conditions.

**Input pathways**: Input pathways provide the core oscillator with input signals to synchronize itself with its surrounding environment, i.e., the change of time of sunrise and sunset throughout the seasons. The following three proteins form the input pathways for *S. elongatus*, and affect its ability to respond to external stimuli: (i) CikA--circadian input kinase: cells that lack cikA have a marked input pathway defect, in that they are unable to to recognize pulses of darkness, and thus cannot reset the phase of their rhythms accordingly. Strains that lack cikA have a shortened circadian period by 2-3 h. CikA is thought to be part of a two-component regulatory system, however the partner response regulator has not been detected. (ii) LdpA--light-dependant period: strains that lack ldpA no longer recognize differences in light intensity as a signal to alter circadian period. The LpdA protein has iron-sulfur clusters, enabling it to sense the redox state of the cell. (iii) Pex--Period extender: binds to the promoter region of KaiA, and is thought to repress KaiA expression. The Pex protein can delay the clock to alter its phase.

**Output pathways**: The circadian clock controls the rhythmic transcription of *S. elongatus *genome. There are two known regulatory mechanisms [6]: *clock-controlled nucleoid compaction *and *molecular activation/repression pathways*. Through direct interaction with KaiC, temporal information flows from the oscillator to SasA. SasA is predicted to activate the response regulator RpaA, of which the target has not been identified. Both SasA and LabA feed information to RpaA, but the connection between LabA and RpaA is probably indirect [6]. Also, another gene named cpmA has been previously described as involving in the output pathway of the cyanobacterial circadian clock, regulating the expression rhythm of kaiA and photosynthesis genes psbAI and psbAII [9].

Although being the subject of intensive studies in the past, there still remain several unanswered questions for *S. elongatus *circadian clock, for example to identify the potential target of the Kai, CikA and RpaA proteins, which can be DNA-binding proteins involved in manipulating compaction of the cyanobacterial chromosome to regulate global gene expression. Another question is whether there are multiple circadian oscillators.

## Results and discussion

### Mapping the circadian clock from S. elongatus to Cyanothece

We start by mapping the clock genes from *S. elongatus *to *Cyanothece*. For this purpose, we used the Cyanobase [10] and BioCyc [11] databases (access June 2012). We used BLAST (with default parameters) to search for the clock genes' homologs in *Cyanothece*. The following Table 1 lists the homologs of the circadian clock genes in the two organisms.

**Table 1:** Mapping of circadian clock genes from *S. elongatus *to *Cyanothece *via Blast search with default parameters.

*S. elongatus*	*Cyanothece*	E-value	***Cyanothece *homolog description (BioCyc **[11])
synpcc7942_1218: kaiA	cce_0424	5e-66	KaiA, circadian clock protein

synpcc7942_1217: kaiB	cce_0423	8e-44	KaiB1, circadian clock protein
	cce_4715	3e-23	KaiB2, putative circadian clock protein
	cce_0435	6e-18	KaiB3, circadian clock protein
	cce_0145	4e-12	KaiB4, putative circadian clock protein

synpcc7942_1216: kaiC	cce_0422	0.0	KaiC1, circadian clock protein
	cce_4716	1e-137	KaiC2, circadian clock protein

synpcc7942_0624: LdpA (light dependent period)	cce_2350	6e-76	putative alpha-helical ferredoxin

synpcc7942_0644: CikA (circadian input kinase)	**cce_4751**	**1e-129**	t**wo-component hybrid sensor and regulator**
	cce_4289	7e-67	two-component hybrid sensor and regulator
	cce_1138	2e-59	two-component hybrid sensor and regulator
	cce_0164	1e-52	two-component sensor histidine kinase
	cce_0220	4e-52	two-component sensor histidine kinase
	cce_2232	3e-46	two-component sensor histidine kinase
	cce_1185	7e-46	two-component hybrid sensor and regulator

synpcc7942_1168: CpmA (circadian phase modifier)	cce_2642	6e-67	circadian phase modifier CpmA-like protein

synpcc7942_0677: Pex (period extender)	-	-	-

synpcc7942_1891: LabA (low-amplitude and bright protein)	**cce_3317**	**7e-78**	**hypothetical protein**
	cce_1947	1e-22	hypothetical protein

synpcc7942_2114: SasA (histidine kinase)	**cce_1751**	**9e-81**	**adaptive-response sensory histidine kinase**
	cce_2546	3e-27	two-component sensor histidine kinase
	cce_0888	4e-25	two-component sensor histidine kinase

synpcc79427942_0095: RpaA (response regulator)	**cce_0298**	**1e-121**	**rpaA two-component response regulator**
	cce_4002	1e-47	rpaB two-component response regulator
	cce_0970	9e-43	two-component transcription regulator
	cce_1725	2e-41	two-component transcriptional regulatory protein
	cce_0817	2e-41	two component transcriptional regulator

The matches with highest E-value are reported.

It can be observed that apart from KaiA which has only a single potential homolog, both KaiB and KaiC have multiple possible homologs, namely KaiB1-4 and KaiC1,2 in *Cyanothece*. The existence of multiple Kai protein suggests the hypothesis that there might be multiple oscillators in *Cyanothece*, of which one might be dedicated to its specialized function of nitrogen fixation. It is noted that for the purpose of this research, we do not differentiate between orthologs and paralogs. Distinguishing orthologs from paralogs is by itself a challenging topic that will be the subject of our future study. LdpA and CpmA also have single homolog in *Cyanothece*. For members of two-component regulatory systems including CikA, SasA and RpaA, multiple possible homologs were found. This result is not surprising, as many cyanobacterial two-component proteins share conserved receptor domains. Herein we list the highest matches according to BLAST. Since the E-value of the best matches generally far exceed those of the other matches, we use only the best matches (bold-face rows in Table 1) as possible homologs. It is worth mentioning that in various databases including Cyanobase [10] and BioCyc [11], the gene cce_4002 was explicitly named as rpaB, suggesting that it has similar function to the rpaA gene. However currently it appears to us that this is likely a computer-based annotation rather than a literature-based annotation. Another notable fact to observe is that there is no Pex homolog in *Cyanothece*. The lack of the Pex protein is also observed with some other cyanobacterial species, suggesting that these organisms must have alternative mechanisms for altering the clock phase which have not yet been discovered.

### Reconstruction of the circadian clock interactions from microarray gene expression data

**Figure 2:**
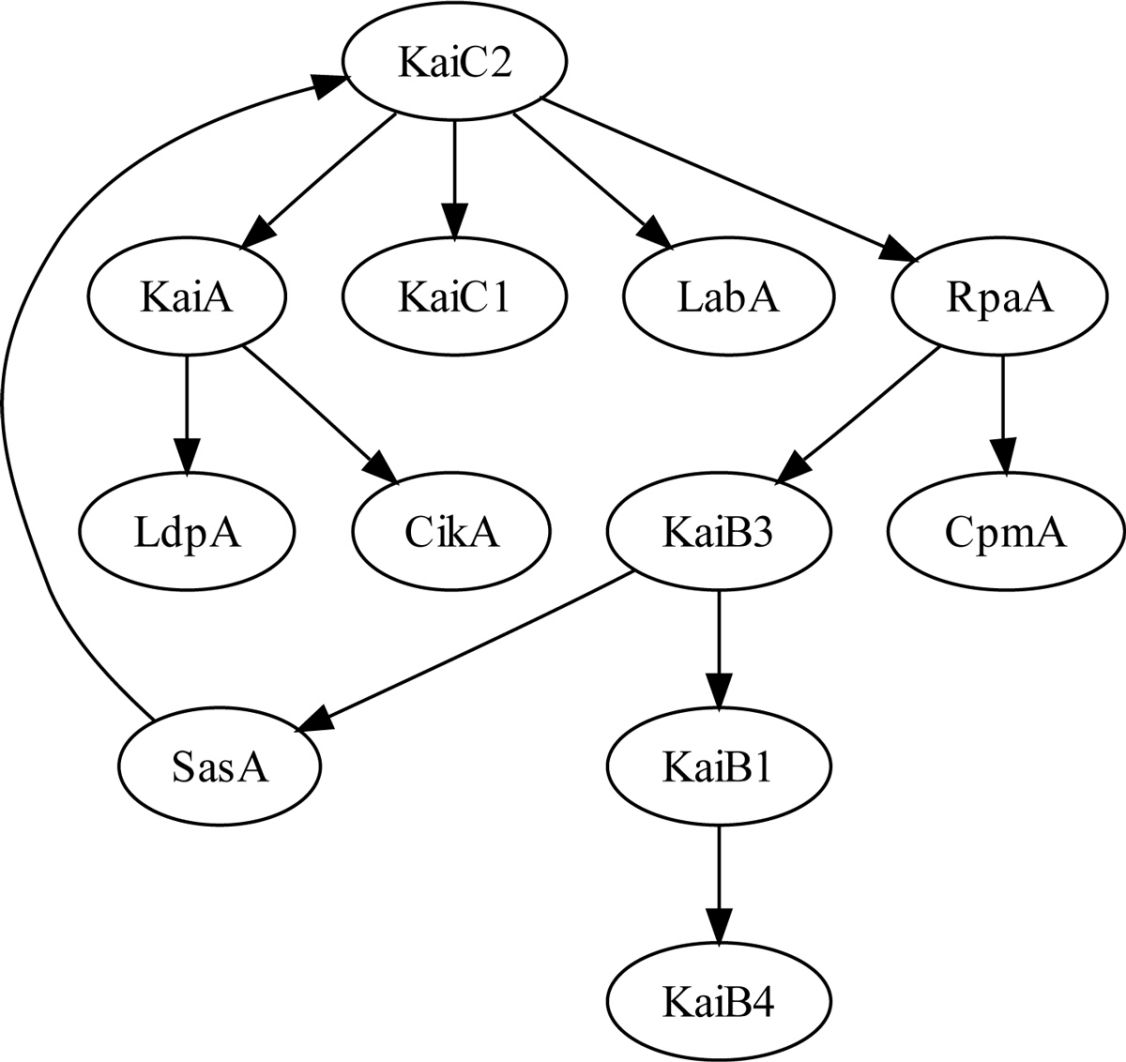
**GlobalMIT^+^-reconstructed *Cyanothece *circadian clock network (for the 12 core clock genes)**. This reconstructed network suggests a central role of the KaiC2 gene within the circadian clock.

Using *Cyanothece *gene expression data for the 12 core clock genes, namely KaiA, KaiB1, KaiB3-4, KaiC1-2, LdpA, CikA, CpmA, LabA, SasA, RpaA, we apply network inference techniques to reconstruct the interactions between these genes (see section 'Data and methods' regarding data collection and preprocessing). The purpose of this study is to investigate to what extent the interactions in *S. elongatus *can be extrapolated to *Cyanothece *as reflected by actual microarray data. The inference tool we employ is our recently developed, dynamic Bayesian network based GlobalMIT^+ ^toolkit [12, 13, see section 'Data and methods']. The microarray data consist of 24 time points corresponding to 3 cycles of 12 h alternative light/dark conditions and 24 h of continuous light condition. Since the sampling rate of 4 h is relatively large compared to common regulation time scale, we used spline interpolation to intrapolate two more data points between each two actual measurements (i.e., upsampling the data at an 1h20' interval). The data were then quantile normalized to three discrete states. GlobalMIT^+ ^was run with the dynamic Bayesian network order set to 1, and the significance parameter *α *= 0.999 as recommended in [12]. It is further noted that apart from the Mutual Information Test (MIT) criterion, GlobalMIT^+ ^also supports the Minimum Description Length (MDL) as the alternative scoring metric. On this particular data set, both metrics returned the same network though, as presented in Figure 2. It is interesting to note that the reconstructed network suggests a central role of the KaiC2 circadian clock gene. Herein KaiC2 is predicted to interact with LabA and RpaA, which belong to the output pathways of *S. elongatus *(Figure 1). This is in concordant with the interactions reported for *S. elongatus*. Another interesting set of connections is KaiC2 → KaiA → {LdpA, CikA}. It can be observed that all the interaction directions are reversed here, as in *S. elongatus*, LdpA and CikA form the input pathways of the clock and interact with KaiA. The KaiC2 → RpaA → CpmA interactions are also notable. It was previously reported that CpmA acts on the output pathway of the circadian clock [9], but whether it is a downstream, upstream or independent gene of RpaA was not reported. For the KaiC2 → RpaA → KaiB3 → SasA→ KaiC2 loop, again it is observed that the directions of interaction are reversed compared to the known network in *S. elongatus*. In particular, it was reported that temporal information flows from KaiC to SaSA to RrpA, but not vice-versa. Overall, we found that the *de novo *reconstructed network using microarray data does shed some light on the *Cyanothece *circadian clock, with some interactions matching those in *S. elongatus*, and poses some novel hypotheses. It remains to verify whether KaiC1 or KaiC2 plays the central role in *Cyanothece *circadian clock, which one is the main oscillator and which one is the peripheral oscillator. The reconstructed network herein suggests KaiC2 to be the central oscillator.

### Building a predictive model of the system behaviour based on the core clock genes

Using Inferelator [14, see section 'Data and methods'], we next build a predictive model between the 12 core clock genes (as regulators) and the other 134 *Cyanothece *putative transcription factors (as targets) which regulate key cellular processes and global transcription. Our aim is first to characterize the cellular processes which are circadian-controlled, and second to see if the constructed model can accurately predict the behaviour of these processes under unknown/changing environmental conditions. For this study, we used 18 time points corresponding to three full 12 h light/dark (L/D) cycles (LDLDLD-with samples taken every 4 h) as our training set. We held out 6 time points corresponding to 24 h of continuous light (LL) as our test set.

#### Training set

**Figure 3:**
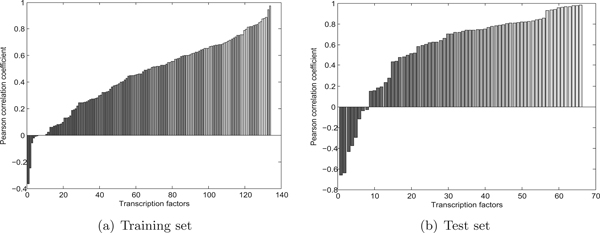
**(a) Correlation for models trained for 134 *Cyanothece *transcription factors on training data; (b) Correlation for models trained for 66 circadian-controlled TFs on test data**.

We run Inferelator, setting the maximum number of single predictors to 5 and interacting predictors to 2, and the time constant *τ *set to 15 mins following previous studies [8,14]. The other parameters were left at default. To validate the constructed model, we employ the leave-one-out cross validation approach as follows: for each given time point, a model is trained using the rest of 17 time points. Then, the value of the left-out time point is predicted as per Eq. (3) assuming steady state condition (Sec. 'Inference methods'). Strictly speaking, our data herein is time series. However, since the sampling gap of 4 h is relatively large compared to the regulation time scale (typically ranging within several tens of minutes [15]), it is reasonable to assume equilibrium condition. This approach was also previously followed in [8] resulting in reasonably good predictions. To assess the quality of the constructed model, we calculate the Pearson correlation coefficient between the predicted and observed time series. The Pearson correlation coefficient for the models trained for 134 *Cyanothece *transcription factors is presented in Figure 3a (ordered in increasing correlation value). The average correlation over all 134 transcription factors (TF) is *ρ *= 0.49. From Figure 3 it is also clear that the circadian clock genes can explain very well the behaviour of a number of TFs, while for the rest of the TFs, the performance is poor using the clock genes as potential regulators alone. This result is to be expected, as *not all the genes and cellular processes are circadian-controlled*. We next apply a threshold of *ρ_min _*= 0.5 to select the TFs that could be regarded circadian-controlled. This filter gives us a set of 66 TFs with the average correlation of 0.68. In Table 2, we list a selected set of circadian-controlled TFs which are well annotated (the rest of these TFs are with fairly vague annotations, such as "two-component response regulator", which are not informative for further analysis). It is noted that in this set, there are the three most important TFs involving in nitrogen fixation, namely ntcA, ntcB and patB. Additionally, the rubisco operon transcriptional regulator rbcR involving in carbon uptake is also presented. Another notable fact to observe is that there is a large number of circadian controlled sigma factors namely rpoD, sigA, sigB, rpoE1, rpoE2, sigD, together with some anti-sigma factors namely cce_0470 and cce_3321. Since it has been previously reported that about 30% of *Cyanothece *genes, i.e., ~1500 genes, are circadian-controlled [7], it is reasonable to expect that the control mechanism is not direct, but indirectly via global chromosome compaction and sigma factors.

**Table 2:** List of transcription factors that are circadian controlled in *Cyanothece*

TF	Training	Testing	Description
cce_0198	0.79	0.75	ntcB nitrogen assimilation transcriptional activator
cce_0461	0.83	0.82	ntcA nitrogen-responsive regulatory protein
cce_1898	0.88	0.58	patB transcriptional regulator (nitrogen fixation)
cce_0470	0.57	0.70	protein containing an Anti-sigma factor antagonist domain
cce_0601	0.65	0.82	rpoD RNA polymerase sigma factor
cce_0644	0.56	0.95	sigB RNA polymerase sigma factor
cce_0875	0.89	0.70	sigA RNA polymerase sigma factor
cce_2424	0.66	0.48	rpoE2 putative RNA polymerase sigma-E factor
cce_2782	0.58	0.74	LysR family transcriptional regulator
cce_2881	0.61	0.81	fur3 ferric uptake regulation protein
cce_3321	0.67	0.77	anti-sigma factor antagonist
cce_3519	0.60	0.60	phoU phosphate uptake regulator
cce_3594	0.75	0.83	sigD RNA polymerase sigma factor
cce_4142	0.72	0.78	rpoE1 RNA polymerase sigma-E factor
cce_3731	0.68	-0.66	rbcR putative Rubisco transcriptional regulator
cce_4701	0.65	0.74	gst3 glutathione S-transferase

#### Test set

**Figure 4:**
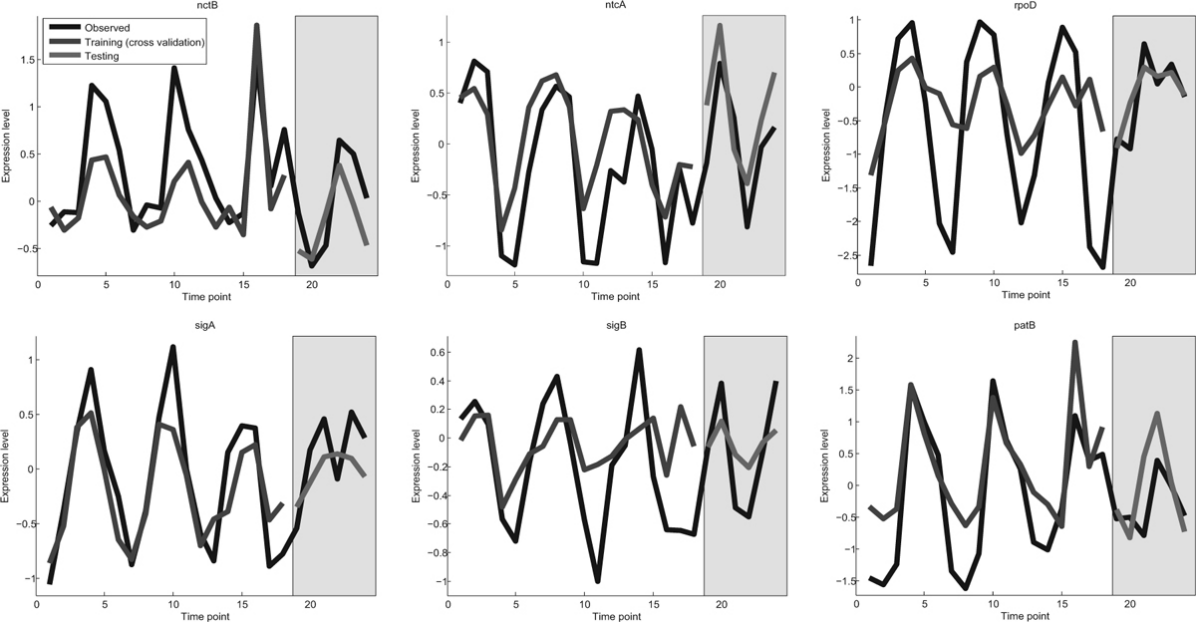
**Observed and predicted expression level for selected TFs**. Yellow bar represents testing conditions. (In gray scale: shaded bar-testing conditions, black line-observed expression, gray line-predicted expression).

Having built a full model for the 66 circadian-controlled TFs on the 18 time-point training set, we next use the hold-out set of 6 time-point data set for validating our model. For this purpose, again we employ Eq. (3) for predicting the values of the target TFs given the values of the core clock genes. The correlation for all 66 TFs and for a selected set of TFs is presented in Figure 3b and Table 2 respectively. The average correlation for all 66 TFs on the test set is 0.57, which is lower than on the training set (0.68). However this result is still quite remarkable, as the test set represents a novel environmental condition (continuous light stress). As from Table 2 and Figure 3b, we can observe that for the majority of the TFs, the learned model can predict very well the system behaviour under this changing environmental condition. The observed and predicted expression levels for some selected TFs are presented in Figure 4. It is noted that while the learned models produce good predictions for the majority of target TFs, the prediction is poor for a small number of TFs, as evidenced in Figure 3b, including the putative Rubisco transcriptional regulator rbcR in Table 2. This is the subject of our future investigation.

## Conclusions

In this paper, we have studied the circadian clock in *Cyanothece *sp. ATCC 51142, an important marine cyanobacterium of high interest in current biofuel research. We have mapped the circadian clock genes and interactions from another species, namely *Synechococcus elongatus *PCC 7942, a model organism for circadian clock study. To validate this extrapolated information, we employed *Cyanothece *gene expression data and network reconstruction techniques. With GlobalMIT^+^, we were able to match some of the genetic interactions in the two organisms and pose some interesting hypotheses, e.g., the central role of KaiC2 in the circadian clock. Using Inferelator, we selected a set of circadian-controlled transcription factors, and built predictive models for these TFs using the core clock genes as regulators. Experimental results showed that our models can fairly accurately predict the behaviour of the system under unknown/changing environmental conditions. There remain for us to answer many more questions regarding the circadian clock in *Cyanothece *as well as in *S. elongatus*, for example identifying the specific targets of many circadian clock genes, including CikA, RrpA and KaiC. In the mean time, our wet-lab experiments are in progress to collect more expression data to supplement the limited *Cyanothece *microarray data currently available in the public domain.

## Data and methods

### Microarray data

Being a relatively under-studied species, currently there are not many microarray data sets available for *Cyanothece*. In this research, we used two data sets that we are aware of from [3] and [16]. In [3]*Cyanothece *cultures were grown in alternative 12 h light/dark (L/D) cycles for 48 h, with samples being collected every 4 h resulting in transcriptomic data for 5048 genes ×12 times points. Using the same experimental protocol, in [16]*Cyanothece *cultures were grown in 12 h L/D cycles for 24 h followed by 24 h of continuous light, with sample being taken every 4 h, also resulting in transcriptomic data for 5048 genes ×12 times points. We downloaded the raw microarray expression data from the European Bioinformatics Institute ArrayExpress http://www.ebi.ac.uk/aerep/, accession number E-TABM-337 and E-TABM-386. The raw microarray intensity values were averaged for probe replicates. Lowess normalization was performed with smoothing coefficient of 0.2. The normalized probe expression values were averaged for biological replicates, technical replicates and dye-swap experiments. Finally, the data were validated to ensure that the median Pearson correlation coefficient is greater for operonic gene pairs than non-operonic gene pairs in *Cyanothece *(using our in-house developed operon prediction tool). To study the core clock and its interaction with key cellular processes and global transcription regulation, in this research, we extracted transcription data for the 12 core clock genes, namely KaiA, KaiB1, KaiB3-4, KaiC1-2, LdpA, CikA, CpmA, LabA, SasA, RpaA (KaiB2 was missing in the above data). In addition, we extracted expression data for 134 other *Cyanothece *putative transcription factors as listed in a recent study [8].

### Inference methods

To reconstruct a regulatory network between the clock genes, we used our in-house recently developed GlobalMIT^+ ^toolkit [12,13]. GlobalMIT^+ ^is a dynamic Bayesian network (DBN) based approach for reconstructing gene regulatory network from time series gene expression data. It is a *score*+*search *based learning technique which employs an information theoretic scoring metric, namely the mutual information test (MIT) criterion. Briefly speaking, under MIT the goodness-of-fit of a network is measured by the total mutual information shared between each node and its parents, penalized by a term which quantifies the degree of statistical significance of this shared information. To understand MIT, let {*r*_1_, ..., *r_n_*} be the number of discrete states corresponding to our set of RVs **X **= {*X*_1_, ..., *X_n_*}, *D *denote our data set of *N *observations, *G *be a DBN, and $Pa_{i}=\left\{X_{i1},.\;.\;.\;,X_{is_{i}}\right\}$ be the set of parents of *X_i _*in *G *with corresponding $\left\{r_{i1},.\;.\;.\;,r_{is_{i}}\right\}$ discrete states, and *s_i _*= |**Pa***_i_*|. The MIT score is defined as:

(1) $$S_{MIT}\left(G:D\right)=\overset{n}{\underset{i=1;Pa_{i}\neq \emptyset }{\sum }}\left\{2N\cdot I\left(X_{i},\;Pa_{i}\right)-\overset{s_{i}}{\underset{j=1}{\sum }}\chi_{\alpha ,l_{i\sigma_{i}\left(j\right)}}\right\}.$$

where *I*(*X_i_*, **Pa***_i_*) is the mutual information between *X_i _*and its parents as estimated from D. $\chi_{\alpha ,l_{ij}}$-is the value such that $p\left(\chi^{2}\left(l_{ij}\right)\leq \chi_{\alpha ,l_{ij}}\right)=\alpha$ (the Chi-square distribution at significance level 1 - *α*), and the term $l_{i\sigma_{i}\left(j\right)}$ is defined as:

$$l_{i\sigma_{i}\left(j\right)}=\left\{\begin{array}{cc} \left(r_{i}-1\right)\left(r_{i\sigma_{i}\left(j\right)}-1\right)\prod_{k=1}^{j-1}r_{i\sigma_{i}\left(k\right)}, & j=2\ldots ,\;s_{i}\\ \left(r_{i}-1\right)\left(r_{i\sigma_{i}\left(j\right)}-1\right), & j=1 \end{array}\right)$$

where *σ_i _*= {*σ_i_*(1), ..., *σ_i_*(*s_i_*)} is any permutation of the index set {1 ... *s_i_*} of **Pa***_i_*, with the first variable having the greatest number of states, the second variable having the second largest number of states, and so on. The prominent features of GlobalMIT^+ ^are its ability to *learn the globally optimal network in polynomial time*, and its competitive performance against other state-of-the-art scoring metrics, such as the Bayesian-Dirichlet (BD) or Bayesian Information Criterion (BIC).

To construct a predictive model for the interaction between the core clock genes and other key process regulators, we employ a well-known differential equation based technique named Inferelator [14]. Differential equation (DE) based approaches are a class of sophisticated, well established methods which have long been used for modeling biochemical phenomena, of which a particularly salient feature is their ability to accurately model the detailed dynamics of biochemical systems in continuous time. Also, unlike DBN based techniques which generally require data discretization, DE-based approaches can work directly with real valued data. Since our aim in this research is also to accurately predict the system behaviour under unknown or changing conditions, a DE-based approach is more suitable for this purpose. In Inferelator, the relation between the expression of a target gene *y *and the expression levels of its regulators *X *is represented as:

(2) $$\tau \frac{\text{d}y}{\text{d}t}=-y+g\left(\beta .Z\right)$$

where *Z *= (*z*_1_(*X*), *z*_2_(*X*), ..., *z_P _*(*X*)) is a set of functions of the regulatory factors *X*, which are in fact either a single variable or the minimum of two variables. *g*(.) is a link function which is chosen to be a truncated linear form, and *τ *is the time constant of the level of *y *in the absence of external determinants. Parameter fitting in Inferelator is done via least angle regression followed by *L*_1 _shrinkage, with cross validation carried out to select parameter values that results in good generalization. To predict the system behaviour assuming equilibrium conditions, setting d*y*/d*t *= 0 we have:

(3) y=g(β.Z)

## Competing interests

The authors declare that they have no competing interests.

## Authors' contributions

All authors conceptualized the research. NXV implemented the algorithms and carried out the experiments. MC provided overall supervision and leadership to the research. NXV and MC drafted the manuscript. RC, SG and PPW suggested the biological data and provided biological insights. All authors read and approved the final manuscript.

## Declarations

The publication costs for this article were funded by the corresponding author's institution.

This article has been published as part of *BMC Bioinformatics *Volume 14 Supplement 2, 2013: Selected articles from the Eleventh Asia Pacific Bioinformatics Conference (APBC 2013): Bioinformatics. The full contents of the supplement are available online at http://www.biomedcentral.com/bmcbioinformatics/supplements/14/S2.

## References

[B1] Bandyopadhyay Anindita Stockel Jana Min Hongtao Sherman Louis A Pakrasi Himadri B High rates of photobiological H2 production by a cyanobacterium under aerobic conditions Nat Commun 2010 1 139 10.1038/ncomms1139 10.1038/ncomms1139 21266989

[B2] Dvornyk Volodymyr Vinogradova Oxana Nevo Eviatar Origin and evolution of circadian clock genes in prokaryotes Proc Natl Acad Sci U S A 2003 100 5 2495 2500 10.1073/pnas.0130099100 12604787 PMC151369

[B3] Stockel Jana Welsh Eric A Liberton Michelle Kunnvakkam Rangesh Aurora Rajeev Pakrasi Himadri B Global transcriptomic analysis of cyanothece 51142 reveals robust diurnal oscillation of central metabolic processes Proc Natl Acad Sci U S A 2008 105 16 6156 6161 10.1073/pnas.0711068105 18427117 PMC2329701

[B4] Nakajima Masato Imai Keiko Ito Hiroshi Nishiwaki Taeko Murayama Yoriko Iwasaki Hideo Oyama Tokitaka Kondo Takao Reconstitution of circadian oscillation of cyanobacterial kaic phosphorylation in vitro Science 2005 308 5720 414 415 10.1126/science.1108451 15831759

[B5] Dong Guogang Golden Susan S How a cyanobacterium tells time Current Opinion in Microbiology 2008 11 6 541 546 Growth and Development: Eukaryotes/Prokaryotes 10.1016/j.mib.2008.10.003 18983934 PMC2692899

[B6] Mackey Shannon R Golden Susan S Winding up the cyanobacterial circadian clock Trends in Microbiology 2007 15 9 381 388 10.1016/j.tim.2007.08.005 17804240

[B7] Wang Wenxue Ghosh BK Pakrasi H Identification and modeling of genes with diurnal oscillations from microarray time series data Computational Biology and Bioinformatics, IEEE/ACM Transactions on 2011 8 1 108 121 jan-feb 10.1109/TCBB.2009.37 21071801

[B8] McDermott Jason E Oehmen Christopher S McCue Lee Ann Hill Eric Choi Daniel M Stockel Jana Liberton Michelle Pakrasi Himadri B Sherman Louis A A model of cyclic transcriptomic behavior in the cyanobacterium cyanothece sp. atcc 51142 Mol BioSyst 2011 7 2407 2418 10.1039/c1mb05006k 21698331

[B9] Katayama Mitsunori Tsinoremas Nicholas F Kondo Takao Golden Susan S cpma, a gene involved in an output pathway of the cyanobacterial circadian system Journal of Bacteriology 1999 181 11 3516 3524 10348865 10.1128/jb.181.11.3516-3524.1999 PMC93820

[B10] Kazusa DNA Research Institute The cyanobacteria database 2011 http://genome.kazusa.or.jp/cyanobase

[B11] Karp Peter D Ouzounis Christos A Moore-Kochlacs Caroline Goldovsky Leon Kaipa Pallavi Ahrén Dag Tsoka Sophia Darzentas Nikos Kunin Victor López-Bigas Núria Expansion of the biocyc collection of pathway/genome databases to 160 genomes Nucleic Acids Research 2005 33 19 6083 6089 10.1093/nar/gki892 16246909 PMC1266070

[B12] Vinh Nguyen Xuan Chetty Madhu Coppel Ross Wangikar Pramod P Gene regulatory network modeling via global optimization of high-order dynamic bayesian network BMC Bioinformatics 2012 13 1 131 10.1186/1471-2105-13-131 22694481 PMC3433362

[B13] Vinh Nguyen Xuan Chetty Madhu Coppel Ross Wangikar Pramod P GlobalMIT: Learning globally optimal dynamic bayesian network with the mutual information test criterion Bioinformatics 2011 27 19 2765 2766 10.1093/bioinformatics/btr457 21813478

[B14] Bonneau Richard Reiss David Shannon Paul Facciotti Marc Hood Leroy Baliga Nitin Thorsson Vesteinn The inferelator: an algorithm for learning parsimonious regulatory networks from systems-biology data sets de novo Genome Biology 2006 7 5 R36 10.1186/gb-2006-7-5-r36 16686963 PMC1779511

[B15] Ramsey Stephen A Klemm Sandy L Zak Daniel E Kennedy Kathleen A Thorsson Vesteinn Li Bin Gilchrist Mark Gold Elizabeth S Johnson Carrie D Litvak Vladimir Navarro Garnet Roach Jared C Rosenberger Carrie M Rust Alistair G Yudkovsky Natalya Aderem Alan Shmulevich Ilya Uncovering a macrophage transcriptional program by integrating evidence from motif scanning and expression dynamics PLoS Comput Biol 2008 4 3 e1000021 10.1371/journal.pcbi.1000021 18369420 PMC2265556

[B16] Toepel Jörg Welsh Eric Summerfield Tina C Pakrasi Himadri B Sherman Louis A Differential transcriptional analysis of the cyanobacterium cyanothece sp. strain atcc 51142 during light-dark and continuous-light growth Journal of Bacteriology 2008 190 11 3904 3913 June 1 10.1128/JB.00206-08 18390663 PMC2395039

